# Investigation of novel material for effective photodegradation of bezafibrate in aqueous samples

**DOI:** 10.1007/s11356-013-2434-y

**Published:** 2014-01-08

**Authors:** Elżbieta Regulska, Joanna Karpińska

**Affiliations:** Department of General and Inorganic Chemistry, Institute of Chemistry, University of Bialystok, Hurtowa 1, 15-399 Bialystok, Poland

**Keywords:** Bezafibrate, Fullerene, Heterogeneous photocatalysis, Nanocomposite, Titanium dioxide

## Abstract

A novel composite with an enhanced photocatalytic activity was prepared and applied to study the removal of bezafibrate (BZF), a hypolypemic pharmaceutical, from an aqueous environment. For the enhancement of titanium dioxide photoactivity a fullerene derivative, 2-(ferrocenyl) fulleropyrrolidine (FcC_60_), was synthesized and applied. Obtained composite was found to show a higher catalytic activity than pristine TiO_2_. Therefore, high hopes are set in composites that are based on carbonaceous nanomaterials and TiO_2_ as a new efficient photocatalysts.

## Introduction

**Scheme 1:**
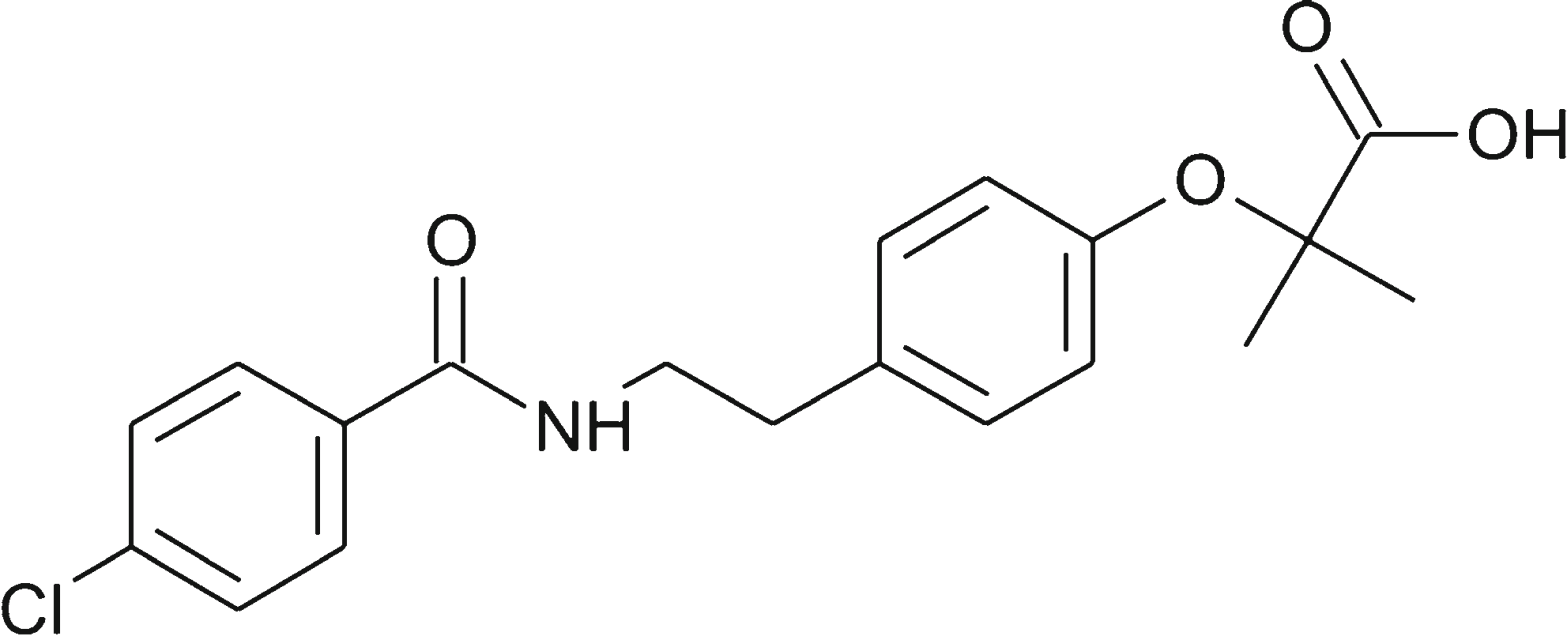
Chemical structure of bezafibrate

Nowadays an occurrence of pharmaceuticals in the aqueous environment is regarded as a serious ecological problem. A variety of drugs were found in the following samples: sewage-treatment plant effluents (Hirsch et al. 1999), surface water (Hirsch et al. 1999; Ternes 1998; Calamari et al. 2003), seawater (Calamari et al. 2003), groundwater (Godfrey et al. 2007) and even in drinking water (Zwiener 2007). Pharmaceuticals are commonly excreted in an unchanged form and/or as metabolites in urine and faeces and discharged into domestic wastewaters continuously. Among innumerable amount of pharmaceuticals dumped into sewage system, bezafibrate (BZF) is classified as persistent contaminant. It is excreted as a parent compound for about 50 % (Castiglioni et al. 2005). Bezafibrate (2-(4-{2-[(4-chlorophenyl)formamido]ethyl}phenoxy)-2-methylpropanoic acid, BZF, Scheme 1), a blood-lipid-lowering agent, is widely used in the treatment of lipidemic diseases such as hypercholesterolemia and hypertriglyceridemia and to prevent heart attack (Weston et al. 2009). Due to the fact that BZF belongs to the most frequently prescribed drugs, it was proved to be ubiquitous in surface water and wastewater (Nikolaou et al. 2007). It was detected in concentrations ranging from 27 ng/L in drinking water (Ternes 2006), 0.1–0.15 μg/L in large rivers (Ternes 2006; Kosma et al. 2007), 0.5–1.9 μg/L in small streams (Ternes 2006), 3.1 μg/L in surface water (Weston et al. 2009) up to 4.6 μg/L level in sewage treatment plant effluents (Weston et al. 2009).

Hence, it is important to estimate efficient methods of BZF removal from a natural aqueous environment. Kinetic studies and identification of degradation products are also of a great concern. Accordingly, those issues were a matter of investigations presented in this paper.

A series of attempts were made in order to adequately remove BZF from natural aqueous samples. They included utilization of flocculation (Ternes et al. 2002), ozonation (Dantasa et al. 2007), filtration with granular activated carbon (Ternes et al. 2002), biodegradation (Kunkel and Radke 2008) and photolysis (Razavi et al. 2009). However, none of them seemed to be sufficiently efficient. Therefore, a huge potential lies in the application of heterogeneous photocatalysis (Lambropoulou et al. 2008). This process has a huge potential as a new route of destruction of organic as well as inorganic hazardous materials (Kabra et al. 2004). Photocatalytic degradation involves a photocatalyst presence. This role is mostly fulfilled by transition metal oxide semiconductors, among which titanium dioxide (TiO_2_) is one of the most widely used. Apart from the removal of pharmaceuticals (Deegan et al. 2011), dyes (Regulska and Karpińska 2012; Regulska et al. 2013; Shang et al. 2011; Fan et al. 2011; Fischer et al. 2011), cyanides (Kabra et al. 2004), malodorous compounds (Kabra et al. 2004), fungicides (Topalov et al. 1999), herbicides (Topalov et al. 2000) and pesticides (Assalin et al. 2010), TiO_2_ was also satisfactorily applied in a destruction of bacteria, viruses (Henderson 2011) and cancer cells (Rozhkova et al. 2009). What is more, due to its photocatalytic activity, TiO_2_ was deeply researched in the field of self-cleaning (Sökmen et al. 2011), self-sterilizing (Hashimoto et al. 2005), highly hydrophilic and anti-fogging surfaces (Hashimoto et al. 2005). Another areas concerning the application of TiO_2_ include dye sensitized solar cells (Jiang et al. 2009), water splitting, CO_2_ photoreduction and the so-called ‘synthesis by photons’ (Arora et al. 2010).

**Scheme 2:**
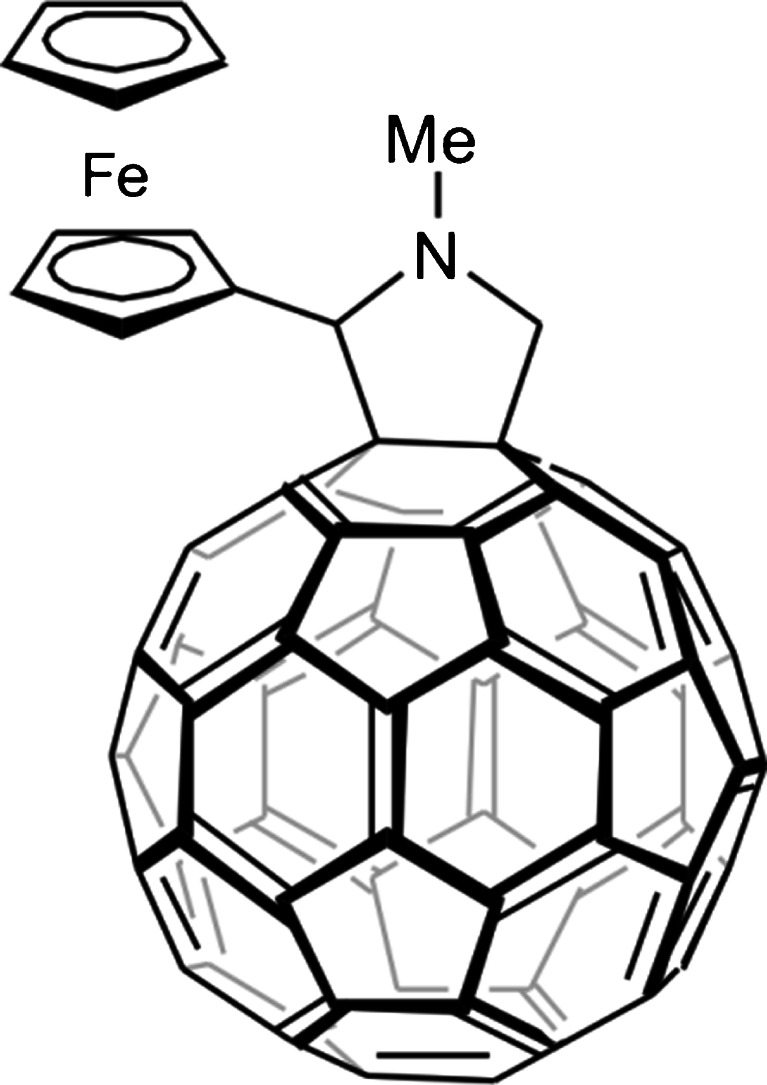
FcC_60_ structure

TiO_2_ owns series of demanding features like low cost, chemical and biological inertness and non-toxicity. Unfortunately, only 3 % of the solar radiation can be used to excite semiconductor molecules (Hashimoto et al. 2005). For this reason, many attempts have been done to enhance TiO_2_ activity (Pan et al. 2010). Among different approaches, including modifying TiO_2_ with charge-transfer catalysts, coating with photosensitizing dyes, noble metal deposition/coupling (Kundu et al. 2011; Zhang et al. 2009), metal doping and grafting, and modifying with polymers (Tomovska et al. 2011) or clays (Arora et al. 2010), carbonaceous nanomaterials (Kochuveedyu et al. 2011; Leary and Westwood 2011; Li et al. 2011; Wang et al. 2012a, 2012b; Yu et al. 2011; Zhang et al. 2011a, 2011b) seem to have the greatest potential. Due to this fact, we have decided to put an effort into preparing and applying a nanocomposite that consisted of titanium dioxide and a fullerene derivative (C_60_), namely 2-(ferrocenyl)fulleropyrrolidine (FcC_60_, Scheme 2). The second constituent of a nanocomposite is a covalently-linked donor–acceptor dyad, which is composed of a fulleropyrrolidine directly linked to the ferrocene (Smith 2006).

So far, no attempts have been made to prepare and examine the above mentioned photocatalyst as a way of enhancing TiO_2_ photocatalytic activity. Therefore, it was described in the present paper. Moreover, the photodegradation process of BZF was studied with the aim of accessing the potential application of prepared photocatalyst in the pharmaceutical removal from the aquatic environment.

## Materials and methods

### Materials

TiO_2_ (anatase), bezafibrate, ferrocenaldehyd, sarcosin and anhydrous toluene were purchased from Sigma-Aldrich. 4-Chlorophenol (CP), 4-chlorobenzoic acid (CBA), 4-hydroxybenzaldehyde (HBD) (all Merck), absolute methanol, dipotassium hydrogen phosphate, orthophosphoric acid (all POCh, Gliwice, Poland) and ammonium reineckate (BDH Chemicals) were used. All above mentioned chemicals were of analytical grade reagents and used without further treatment. HPLC-grade acetonitrile was purchased from J.T. Baker. All solutions were prepared using deionized water, which was obtained by Polwater apparatus.

### Apparatus

Scanning electron microscopy was applied to investigate the morphology of the FcC_60_/TiO_2_ composite. Composite was imaged by secondary electron SEM with the use of an Inspect S50 scanning electron microscope from FEI. The accelerating voltage of the electron beam was 30 keV and the working distance was 10 mm. Differential scanning calorimetry (DSC) analyses were performed by a thermal analyzer DSC 1 (Mettler Toledo) with a heating rate of 10 °C/min under air environment with flow rate = 200 mL/min. All runs were carried out from 0 to 500 °C and reverse cycles from 500 to 0 °C were registered too. The measurements were made in open aluminium crucibles, nitrogen was used as the purge gas in ambient mode, and calibration was performed using an indium standard. Attenuated total reflection infrared (ATR-IR) spectra were recorded from 4,000 to 500 cm^−1^ using a Thermo Scientific Nicolet™ 6700 spectrometer with 32 accumulations at a resolution of 4 cm^−1^. This instrument was equipped with a KBr beamsplitter, an ETC source and a DTGS detector. Photolytic as well as photocatalytic degradation experiments were carried out in a solar simulator apparatus, namely SUNTEST CPS+ (Atlas, USA). The photon flux of solar simulated radiation was measured by chemical method—Reinecke’s salt actinometer (Kuhn et al. 2004). The photon flux of solar simulated light of 250 W/m^2^ was 2.18 × 10^−6^ Einstein/s. The chromatographic experiments with HPLC–UV system were carried out on a Varian 920 liquid chromatograph using a quaternary solvent pump and an autosampler. The chromatographic column used Lichrospher®100 RP-18 125 × 4.6 mm packed with 5 μm particle size. Separation was achieved using a linear gradient method. The mobile phase consisted of two solutions namely A and B. Solution A was prepared from 10 mmol/L disodium hydrogen phosphate, pH adjusted to 7.4 with orthophosphoric acid, whereas solution B was acetonitrile. The initial ratio of A:B was 70:30 (*v*/*v*). The gradient was as follows: 0 min, 70 % A; 15 min, 70 % A; 25 min, 20 % A; 30 min, 20 % A; and 35 min, 70 % A. The column was equilibrated for 10 min before performing the next injection. The flow rate of the mobile phase was 1 mL/min and the injection volume was 100 μL. The column was maintained at a room temperature. The eluent was monitored at 226 nm. UV spectrophotometric analyses were performed in a Hitachi U-2800A UV–Vis spectrophotometer equipped with a double monochromator and double beam optical system (190–700 nm). UV studies were done using a 1-cm quartz cell. Absorbance was recorded in the range of 190–400 nm, and the maximum absorption wavelength experimentally registered at *λ* = 226 nm was used for the calibration curve and further concentration measurements.

### Photocatalyst preparation

FcC_60_ was synthesized according to the following procedure. A solution of 100 mg of C_60_, 60 mg of ferrocene aldehyde and 25 mg of N-methyl glycine in 100 mL of toluene was stirred at reflux temperature overnight. The solvent was removed in vacuum. The solid residue was purified by flash chromatography using toluene as an eluent affording 81 mg (21 %). ^1^H NMR (400 MHz, CDCl_3_:CS_2_ = 1:1 *v*/*v*) δ 3.05 (s, 3H), 4.20 (m, 1H), 4.21 (m, 1H), 4.26 (m, 1H), 4.28 (s, 1H), 4.30 (s, 4H), 4.48 (m, 1H), 4.49 (s, 1H), 4.58 (m, 1H), 4.84 (m, 1H), 4.88 (s, 1H), and 4.91 (s, 1H); ^13^C NMR (100 MHz, CDCl_3_:CS_2_ = 1:1 *v*/*v*) 41.76, 67.02, 67.12, 67.56, 68.21, 69.31, 70.95, 77.00, 141.82, 142.37, and 145.67.

FcC_60_/TiO_2_ nanocomposite was prepared by evaporation–drying method (Yao et al. 2008), previously applied to prepare carbon nanotubes–TiO_2_ nanocomposites. FcC_60_/TiO_2_ composite was made at 1:20 mass ratio of FcC_60_ to TiO_2_.

### Photocatalytic degradation experiment

The photocatalytic degradation experiments were performed in a 50-mL glass cell. The reaction mixture consisted of 20 mL of BZF sample (5 × 10^−5^ mol/L) and the photocatalyst (1.6 g/L). The pharmaceutical-catalyst suspension was kept in the dark with stirring for 1 h to ensure an adsorption–desorption equilibrium prior to the irradiation experiment. Such prepared mixture was subjected to irradiation by simulated sunlight for 120 min. To determine the BZF degradation, the samples were collected at regular intervals (10 min) and centrifuged to remove the photocatalyst and the spectra of the obtained solution were recorded.

## Results and discussion

The characterization of synthesized composite was done before photocatalysis experiments. For this purpose scanning electron microscopy, DSC and ATR-IR spectrometry were used.

**Fig. 1:**
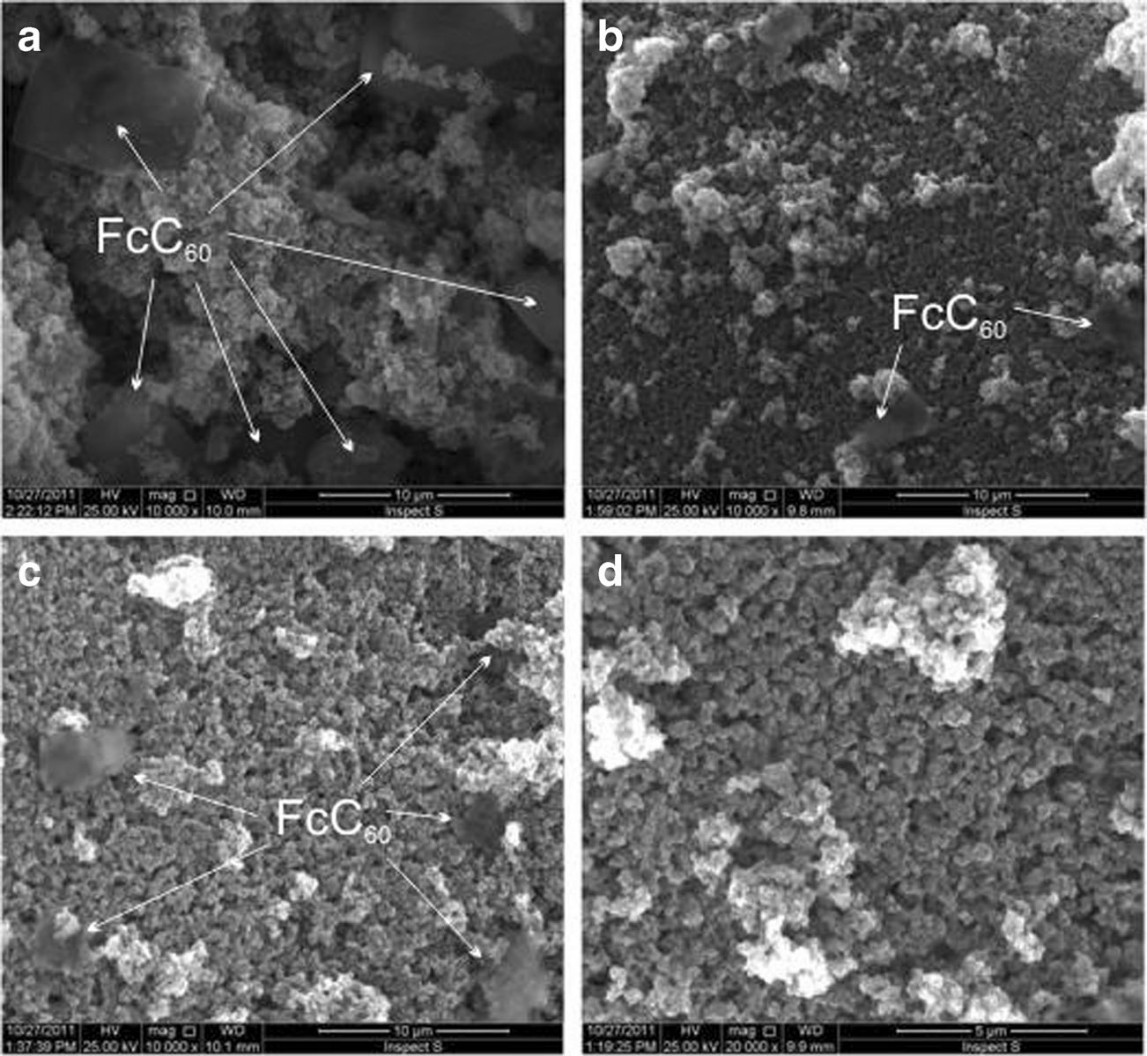
SEM images of FcC_60_/TiO_2_ in the mass ratios of 1:1 (*a*), 1:10 (*b*), 1:20 (*c*) and TiO_2_ (*d*)

Scanning electron microscopy was employed to investigate the morphology of the prepared FcC_60_/TiO_2_ composite. SEM images of pristine TiO_2_ and composites of different mass ratios (1:1, 1:10 and 1:20) of FcC_60_ to TiO_2_ were presented in Fig. 1. They show the presence of both components of the prepared material. The larger flakes with a smooth surface correspond to the FcC_60_ while the smaller particles to the TiO_2_. The proportions of smooth flakes to the smaller particles on presented pictures are in agreement with the mass ratios of FcC_60_ to TiO_2_ in the prepared composites. It can be seen that the greater the amount of TiO_2_ was introduced, the smaller smooth surface is present in the image. It can be attributed to the coverage of carbonaceous surface by TiO_2_ particles.

**Fig. 2:**
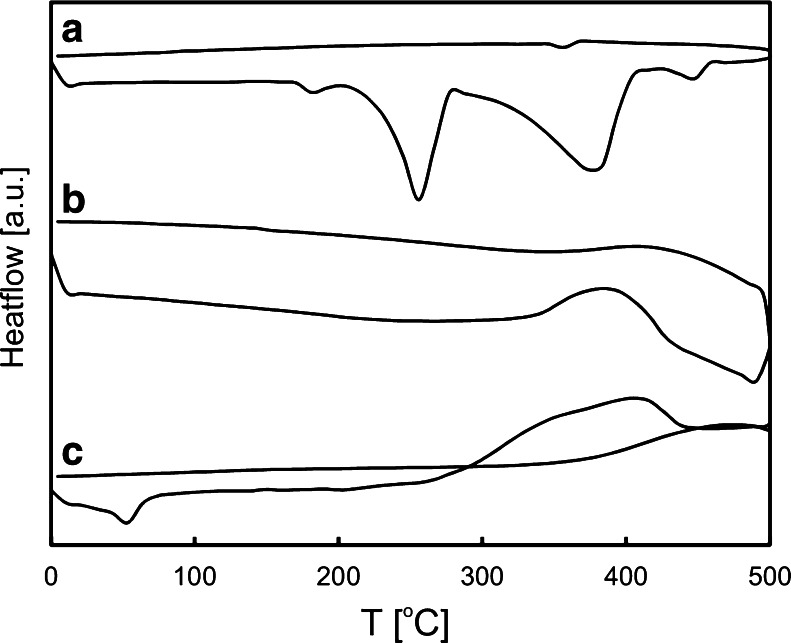
DSC curves of BZF (*a*), FcC_60_/TiO_2_ (*b*) and FcC_60_/TiO_2_ after adsorption of BZF (*c*)

DSC curves of BZF, FcC_60_/TiO_2_ and FcC_60_/TiO_2_ after adsorption of BZF were registered (Fig. 2). The DSC curve of BZF showed a sharp endothermic peak at 187 °C, which is typical for crystalline form α of that pharmaceutical (Lemmerer et al. 2009). BZF does not show any crystallization exotherms upon cooling. However, above mentioned peak representing BZF melting is not observed on the DSC curve of FcC_60_/TiO_2_ after adsorption of BZF without further irradiation. Therefore, the DSC curve of FcC_60_/TiO_2_ after adsorption of BZF differs from the DSC curve of a bare FcC_60_/TiO_2_. That indicates the modification of the photocatalyst surface as a consequence of a pharmaceutical adsorption. It should be mentioned, that the major changes between the first and the third curve may be a consequence of the different values of heat flow in FcC_60_/TiO_2_ and BZF. Photocatalyst is resistant to temperature influence in applied temperature ranges.

**Fig. 3:**
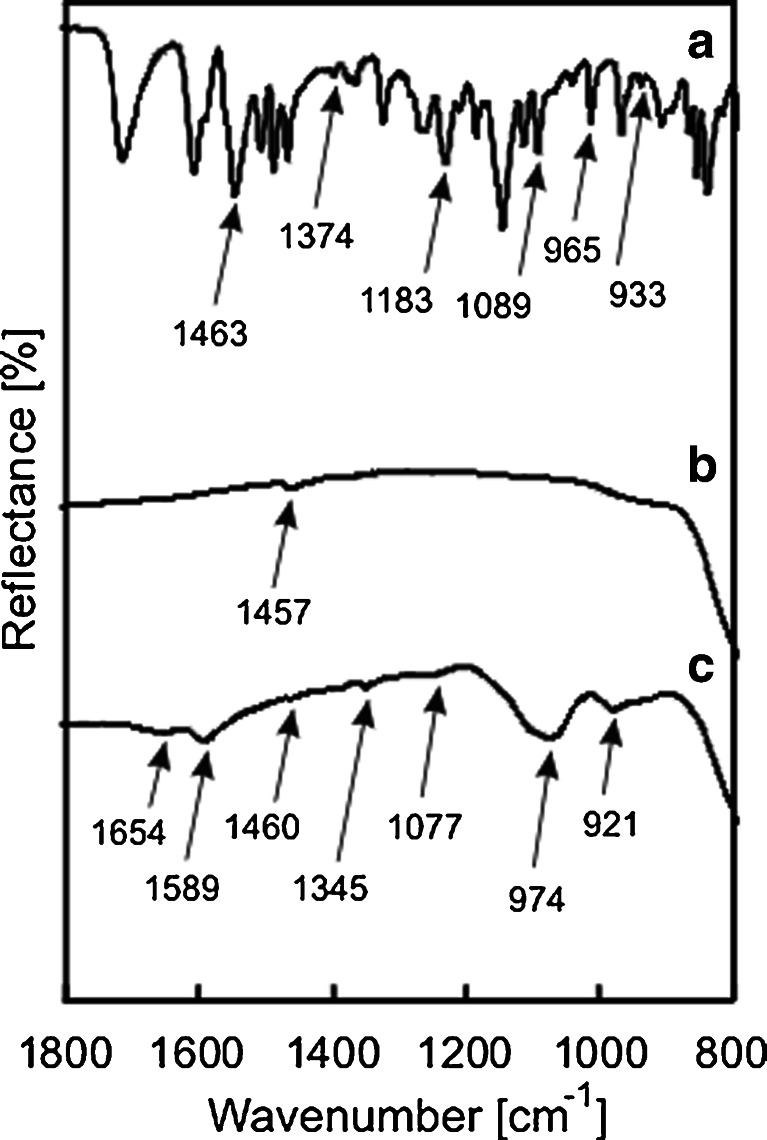
ATR-IR spectra of BZF (*a*), FcC_60_/TiO_2_ (*b*) and FcC_60_/TiO_2_ after adsorption of BZF (*c*)

ATR-IR spectra of BZF, FcC_60_/TiO_2_ and FcC_60_/TiO_2_ after adsorption of BZF were compared and presented in Fig. 3. Spectra were presented in the range of wavelengths in which the biggest differences were observed. After adsorption of BZF on the FcC_60_/TiO_2_ surface certain bands, namely at 1,589, 1,077, 974 and 921 cm^−1^, appeared. The band at 1,589 cm^−1^ was ascribed to the N–H bending vibration ν_N–H_ of secondary amines, while the band at 1,077 cm^−1^ was assigned to the stretching vibrations of the bond between the aromatic carbon and chlorine (C_Ar–Cl_) as well as to that of the ether group C–O–C. Those results confirm modification of the photocatalyst surface after the adsorption of BZF.

**Fig. 4:**
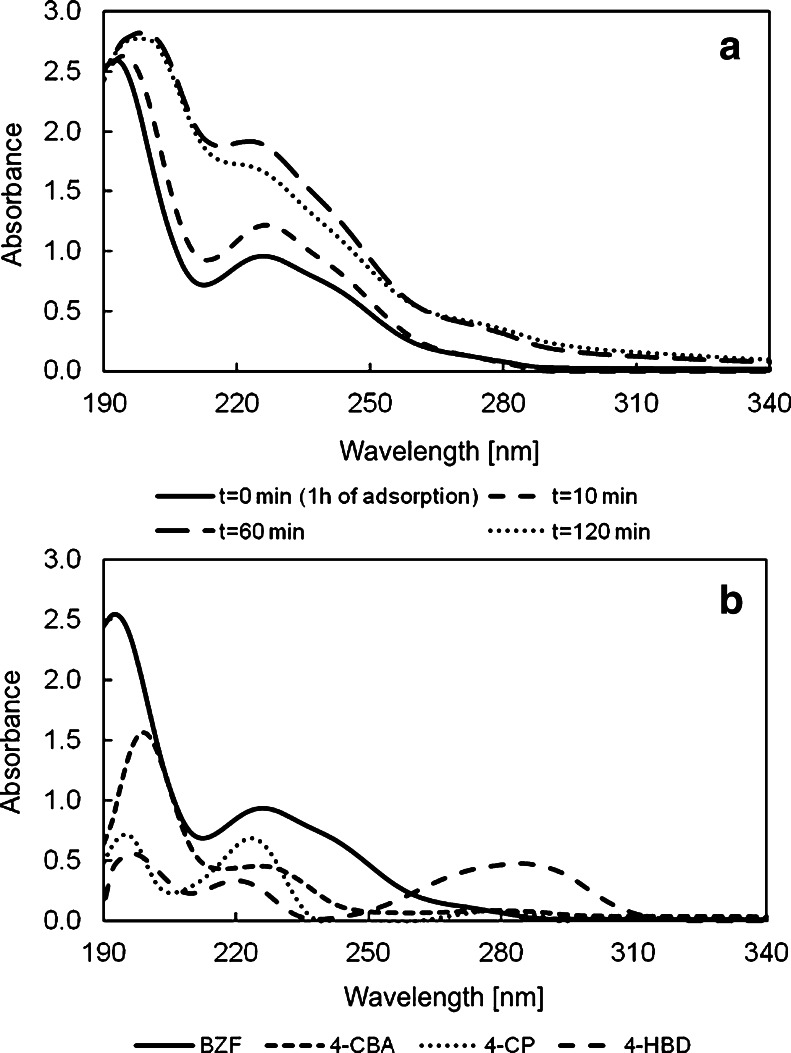
Spectra of BZF before (*t* = 0 min) and after photocatalysis (*t* = 10, 60, 120 min) (**a**). UV absorbance spectra of BZF, 4-CBA, 4-CP and 4-HBD (**b**). *BZF* bezafibrate, *4-CBA* 4-chlorobenzoic acid, *4-CP* 4-chlorophenol, *4-HBD* 4-hydroxybanzaldehyde

UV spectrophotometry was applied to monitor the absorbance changes during irradiation of BZF in FcC_60_/TiO_2_ suspension with solar simulated light. An increase in the absorbance values was observed (Fig. 4a). However, no additional peaks were found in the registered spectra. This fact suggests that BZF does not undergo mineralization or its decomposition with the creation of photoproducts which absorb at the same UV region takes place.

**Fig. 5:**
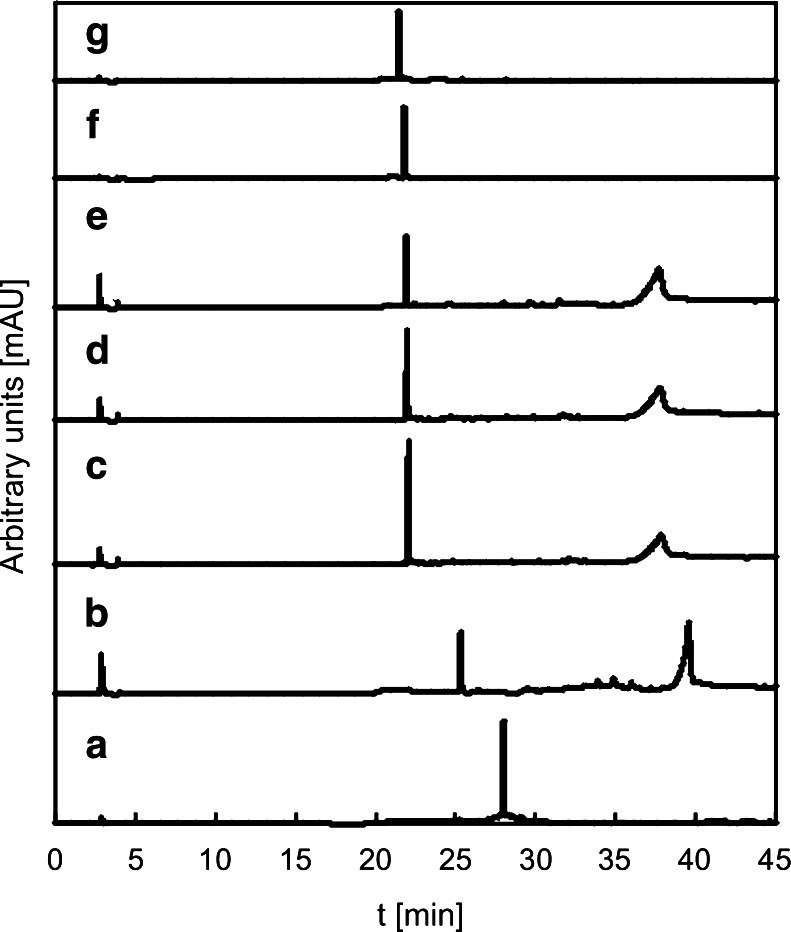
Chromatograms of BZF before irradiation (*a*) and after 10 (*b*) and 120 min of photolysis (*c*) as well as photocatalysis with application of both TiO_2_ (*d* 10 min, *e* 120 min) and FcC_60_/TiO_2_ (*f* 10 min, *g* 120 min)

HPLC analysis was used to confirm above assumption. Chromatograms of BZF before irradiation and after 10 and 120 min of photolysis (a–c in Fig. 5), as well as photocatalysis with application of both TiO_2_ (d and e in Fig. 5) and FcC_60_/TiO_2_ photocatalysts (f and g in Fig. 5), were recorded and compared.

The obtained chromatograms proved that BZF undergoes photodegradation upon solar simulated light with the use of FcC_60_/TiO_2_ as well as TiO_2_ alone. FcC_60_/TiO_2_ nanocomposite was found to be more efficient photocatalyst than bare TiO_2_. In solutions with the presence of FcC_60_/TiO_2_ after only 10 min of irradiation remained only the one final photodegradation product (*t*_r_ = 22.0). During photocatalytic decomposition of BZF with TiO_2_ application even after 2 h of exposure to the solar simulated light still remained four pharmaceutical degradation products. Compounds with their retention times detected by HPLC method were presented in Table 1. Three products of photocatalytic decomposition of BZF were identified using standard references. The obtained results are in agreement with the mechanism of BZF photodegradation proposed by Lambropoulou et al. (2008). Those compounds include 4-hydroxybanzaldehyde (4-HBD), 4-chlorophenol (4-CP) and 4-chlorobenzoic acid (4-CBA). This outcome explains why no changes were observed in the spectra registered during photocatalytic experiments after irradiation. It was found that spectra of identified photodegradation products and bezafibrate are strongly overlapped (Fig. 4b). The increase in amount of generated photoproducts resulted in the increase of absorbance of final mixture. What is more, those compounds have higher absorption coefficients; therefore, during irradiation in the photocatalyst presence, increase in the absorbance is observed.

**Table 1:** Identified compounds by HPLC analysis

Compound	Name	Retention time [min]	Photolysis (120 min)	Photocatalysis (TiO_2_) (120 min)	Photocatalysis (FcC_60_/TiO_2_) (120 min)
1	4-Hydroxybanzal-dehyde	4.0	+	+	−
2	4-Chlorophenol	22.0	+	+	+
3	Bezafibrate	27.7	−	−	−
4	4-Chlorobenzoic acid	37.7	+	+	−

## Conclusions

The efficiency of photocatalytic activity of newly synthesized composite was proved. BZF was found to undergo complete decomposition with the application of both TiO_2_ and FcC_60_/TiO_2_. It was noticed that in the presence of FcC_60_/TiO_2_ bezafibrate is quickly transformed into 4-CP instead of three (4-HBD, 4-CP, 4-CBA) in case of photolytic and photocatalytic degradation with the application of bare TiO_2_.

Conducted experiments confirmed photocatalytic ability of FcC_60_/TiO_2_ composite. What is more, prepared photocatalyst showed higher catalytic activity than sole TiO_2_. Therefore, we believe that huge potential lies in composites that are based on carbonaceous nanomaterials and TiO_2_ as new efficient photocatalysts. Prepared composite could potentially be used in the decontamination of other organic pollutants from water. Heterogeneous catalysis was proved to have a huge potential as a new route of a destruction of undesired compounds present in the environment. However, the photocatalyst application should be preceded by preliminary studies concerning an identification of degradation products and an assessment of their toxicity.
